# Supplementary material for: Behavioral entrainment to rhythmic auditory stimulation can be modulated by tACS depending on the electrical stimulation field properties
Source: eLife. 2024 Jan 30;12:RP87820. doi: 10.7554/eLife.87820 (PMC10945705; doi:10.7554/eLife.87820)
Supplement: Supplementary file 1. — (a). MNI center coordinates for target functional regions of interest for each subject. (b) Statistics for the mixed effects logistic regression models predicting single trial gap detection performance. Models are organized from smallest to highest AIC. Δ AIC relative to winning model. The winning model is also highlighted in bold. BIC: Bayesian information criterion. (c) General linear models predicting inter-session difference on tACS-amplitude. Models are organized from smallest to highest AICc. * Δ AICc relative to winning model. The winning model is also highlighted in bold. (d) General linear models predicting inter-session absolute circular distance on tACS-phase. Models are organized from smallest to highest AICc. * Δ AICc relative to winning model. The winning model is also highlighted in bold. (e) General linear models predicting tACS effects. Models are organized from smallest to highest AICc. * Δ AICc relative to winning model. The winning model is also highlighted in bold. [file elife-87820-supp1.docx]

**Supplementary file 1a**

| Subject | Right hemisphere (x. y. z) | Left Hemisphere (x. y. z) |
| --- | --- | --- |
| 1 | 56.328. -23.081. 5.419 | -53.954. -28.559. 8 |
| 2 | 50.722. -18.320. 7.010 | -54.714. -15.714. 6.286 |
| 3 | 61.478. -21.304. 7.565 | -56.820. -21.533. 5.072 |
| 4 | 57.482. -20.392. 1.396 | -53.491. -25.509. 4.541 |
| 5 | 58.336. -15.748. 4.899 | -54.254. -22.254. 5.559 |
| 6 | 56.226. -9.2260. 1.516 | -51.692. -10.462. 0.385 |
| 7 | 55.907. -25.389. 7.130 | -51.268. -29.895. 9.579 |
| 8 | 62.769. -22. 10.077 | -63.571. -23.571. 6.429 |
| 9 | 55.442. -17.731. 6.673 | -56.679. -22.643. 8.911 |
| 10 | 59.404. -29.527. 4.567 | -54.790. -25.559. 7.378 |
| 11 | 55.935. -15.613. 0.1610 | -51.500. -20.357. 1.286 |
| 12 | 54.150. -16.750. 3.500 | -57.343. -24.057. 9.114 |
| 13 | 52.759. -12.328. 2.724 | -48.783. -24.832. 9.081 |
| 14 | 58.917. -16.887. 3.702 | -54.884. -28.231. 6.787 |
| 15 | 52.340. -17.814. 3.633 | -50.180. -30.329. 10.273 |
| 16 | 54.368. -22.263. 3.737 | -48.278. -24.845. 7.320 |
| 17 | 58.643. -25.881. 9.238 | -54.026. -28.416. 7.675 |
| 18 | 57.471. -22.422. 7.922 | -54.475. -27.050. 8.307 |
| 19 | 58.098. -22.108. 5.647 | -53.211. -23.376. 7.193 |
| 20 | 62.803. -10.039. 3.618 | -49.769. -22.846. 9.462 |
| 21 | 60.239. -25.271. 3.401 | -56.011. -22.379. 4.629 |
| 22 | 52.548. -26.839. 13.226 | -57.951. -19.951. 5.659 |
| 23 | 50.070. -18.366. 5.127 | -50.029. -25.441. 8.971 |
| 24 | 55.563. -19.403. 7.672 | -54.150. -27.175. 6.950 |
| 25 | 58.549. -11.310. 1.028 | -52.122. -20.204. 5.571 |
| 26 | 53.714. -24.333. 6.952 | -51.435. -27.355. 6.419 |
| 27 | 59.127. -23.131. 7.138 | -51.872. -24.702. 6.830 |
| 28 | 47.600. -28.600. 5 | -55.721. -25.397. 9.588 |
| 29 | 59.524. -20.452. 3.595 | -60.577. -20.731. 6.154 |
| 30 | 54.923. -13.231. -1.462 | -55.589. -27.881. 9.144 |
| 31 | 53.910. -18.594. 4.075 | -52.071. -33.614. 10.614 |
| 32 | 57.364. -26.150. 8.196 | -61.533. -25.800. 7.467 |
| 33 | 55.762. -22.992. 7.053 | -53.278. -26.711. 9.422 |
| 34 | 54.862. -21.552. 8.862 | -50.090. -24.426. 7.761 |
| 35 | 58.468. -25.152. 3.949 | -55.136. -23.412. 6.038 |
| 36 | 58.347. -23.042. 6.627 | -53.846. -25.949. 7.360 |
| 37 | 62.104. -20.007. 5.582 | -54.661. -18.835. 4.009 |
| 38 | 57.733. -23.168. 5.426 | -51.351. -28.511. 10.218 |
| 39 | 59.427. -22.708. 6.6850 | -54.-27.407. 9.037 |

**Supplementary file 1b**

|  | Formula | AIC | Δ AIC | BIC | LogLikelihood | Deviance |
| --- | --- | --- | --- | --- | --- | --- |
| 1 | ***Gap detection* ~ 1 + sinFM + cosFM + (1 + sinFM + cosFM \| participant)** | **46560** | **0** | **46637** | **-23271** | **46542** |
| 2 | *Gap detection* ~ 1 + sintACS + costACS + sinFM + cosFM + (1 + sintACS + costACS + sinFM + cosFM \| participant) | 46566 | 6.203 | 46738 | -23263 | 46526 |
| 3 | *Gap detection* ~ 1 + sintACS*sinFM + costACS*sinFM + sintACS*cosFM + costACS*cosFM + (1 + sintACS:sinFM + costACS:sinFM + sintACS:cosFM + costACS:cosFM \| participant) | 47252 | 691.635 | 47457 | -23602 | 47204 |

**Supplementary file 1c**

|  | Formula | AICc | Δ AICc* |
| --- | --- | --- | --- |
| **1** | ***ΔtACS-amplitude* ~ 1 + ΔDays + ΔMinutes** | **97.182** | **0** |
| 2 | *ΔtACS-amplitude* ~ 1 + Age + ΔDays + ΔMinutes | 97.310 | 0.129 |
| 3 | *ΔtACS-amplitude* ~ 1 + Age + ΔDays + ΔMinutes + Δgap size threshold | 97.629 | 0.447 |
| 4 | *ΔtACS-amplitude* ~ 1 + Age + ΔDays + ΔMinutes + Δgap size threshold + Montage | 100.047 | 2.866 |
| 5 | *ΔtACS-amplitude* ~ 1 ΔDays | 100.289 | 3.107 |
| 6 | *ΔtACS-amplitude* ~ 1 + ΔMinutes | 101.269 | 4.087 |
| 7 | *ΔtACS-amplitude* ~ 1 + Gender + Age + ΔDays + ΔMinutes + Δgap size threshold + Montage | 102.803 | 5.621 |

**Supplementary file 1d**

|  | Formula | AICc | Δ AICc* |
| --- | --- | --- | --- |
| **1** | ***\|circ_distance\|* ~ 1 + gender** | **102.416** | **0** |
| 2 | *\|circ_distance\|* ~ 1 + gender + Δgap size threshold | 102.703 | 0.288 |
| 3 | *\|circ_distance\|* ~ 1 + gender + ΔMinutes + Δgap size threshold | 103.285 | 0.869 |
| 4 | *\|circ_distance\|* ~ 1 + gender + Age + ΔMinutes + Δgap size threshold | 104.498 | 2.083 |
| 5 | *\|circ_distance\|* ~ 1 + gender + Age + ΔMinutes + Δgap size threshold + Montage | 106.564 | 4.148 |
| 6 | *\|circ_distance\|* ~ 1 + gender + Age + ΔDays + ΔMinutes + Δgap size threshold + Montage | 109.663 | 7.247 |

**Supplementary file 1e**

|  | Formula | AICc | Δ AICc* |
| --- | --- | --- | --- |
| **1** | ***tACS_amplitude* ~1 + Dist2Peak*Normal_E_field + Normal_E_field*Focality** | **94.429** | **0** |
| 2 | tACS_amplitude ~1 + Corr2BOLD*Normal_E_field_ROI + Normal_E_field_ROI*Focality | 95.691 | 1.262 |
| 3 | tACS_amplitude ~1 + Corr2BOLD*E_field_ROI + E_field_ROI*Focality | 95.853 | 1.423 |
| 4 | tACS_amplitude ~1 + Corr2BOLD*Normal_E_field + Normal_E_field*Focality | 96.719 | 2.289 |
| 5 | tACS_amplitude ~1 + Normal_E_field*Focality | 97.127 | 2.697 |
| 6 | tACS_amplitude ~1 + Dist2Peak*E_field + E_field*Focality | 97.156 | 2.727 |
| 7 | tACS_amplitude ~1 + Dist2Peak*Normal_E_field + Dist2Peak*Focality + Normal_E_field*Focality | 97.535 | 3.105 |
| 8 | tACS_amplitude ~1 + E_field*Focality | 97.654 | 3.225 |
| 9 | tACS_amplitude ~1 + Corr2BOLD*E_field + E_field*Focality | 97.727 | 3.297 |
| 10 | tACS_amplitude ~1 + Dist2Peak*Normal_E_field_ROI + Normal_E_field_ROI*Focality | 98.022 | 3.592 |
| 11 | tACS_amplitude ~1 + Dist2Peak*Normal_E_field_ROI + Dist2Peak*Focality + Normal_E_field_ROI*Focality | 98.347 | 3.917 |
| 12 | tACS_amplitude ~1 + Corr2BOLD*Normal_E_field' | 98.463 | 4.034 |
| 13 | tACS_amplitude ~1 + Dist2Peak*Focality + Normal_E_field_ROI*Focality | 99.191 | 4.761 |
| 14 | tACS_amplitude ~1 + Dist2Peak*E_field + Dist2Peak*Focality + E_field*Focality | 99.423 | 4.993 |
| 15 | tACS_amplitude ~1 + Corr2BOLD*Normal_E_field_ROI | 99.838 | 5.409 |
| 16 | tACS_amplitude ~1 + Corr2BOLD*E_field | 99.852 | 5.422 |
| 17 | tACS_amplitude ~1 + Dist2Peak*Focality + Normal_E_field*Focality' | 100.287 | 5.858 |
| 18 | tACS_amplitude ~1 + Sham + BOLDbetaMean + Corr2BOLD*E_field_ROI + E_field_ROI*Focality | 100.538 | 6.109 |
| 19 | tACS_amplitude ~1 + Dist2Peak*E_field_ROI + E_field_ROI*Focality | 100.993 | 6.564 |
| 20 | tACS_amplitude ~1 + Sham + BOLDbetaMean + Corr2BOLD*Normal_E_field_ROI + Normal_E_field_ROI*Focality' | 101.048 | 6.618 |
| 21 | tACS_amplitude ~1 + Dist2Peak*E_field_ROI + Dist2Peak*Focality + E_field_ROI*Focality | 101.629 | 7.200 |
| 22 | tACS_amplitude ~1 + Dist2Peak*Focality + E_field*Focality | 101.696 | 7.267 |
| 23 | tACS_amplitude ~1 + Dist2Peak*Focality + E_field_ROI*Focality | 101.785 | 7.356 |
| 24 | tACS_amplitude ~1 + Sham + BOLDbetaMean + Corr2BOLD*Normal_E_field + Normal_E_field*Focality | 102.473 | 8.043 |
| 25 | tACS_amplitude ~1 + Sham + BOLDbetaMean + Corr2BOLD*E_field + E_field*Focality | 102.796 | 8.366 |
| 26 | tACS_amplitude ~1 + Dist2Peak*Normal_E_field_ROI + Dist2Peak*Focality | 103.616 | 9.187 |
| 27 | tACS_amplitude ~1 + Sham + BOLDbetaMean + Dist2Peak*Normal_E_field + Dist2Peak*Focality + Normal_E_field*Focality | 104.304 | 9.875 |
| 28 | tACS_amplitude ~1 + Dist2Peak*E_field + Dist2Peak*Focality | 104.308 | 9.879 |
| 29 | tACS_amplitude ~1 + Sham + BOLDbetaMean + Dist2Peak*Normal_E_field_ROI + Dist2Peak*Focality + Normal_E_field_ROI*Focality | 104.439 | 10.010 |
| 30 | tACS_amplitude ~1 + Dist2Peak*Normal_E_field + Dist2Peak*Focality | 104.844 | 10.415 |
| 31 | tACS_amplitude ~1 + Dist2Peak*E_field_ROI + Dist2Peak*Focality | 105.275 | 10.845 |
| 32 | tACS_amplitude ~1 + Sham + BOLDbetaMean + Dist2Peak*E_field + Dist2Peak*Focality + E_field*Focality | 105.285 | 10.856 |
| 33 | tACS_amplitude ~1 + Sham + BOLDbetaMean + Dist2Peak*E_field_ROI + Dist2Peak*Focality + E_field_ROI*Focality | 106.965 | 12.535 |
